# Biosecurity practices in the dairy farms of southern Brazil

**DOI:** 10.3389/fvets.2024.1326688

**Published:** 2024-03-27

**Authors:** Janaína Santos Ferreira, Camila Costa Baccili, Beatriz S. Nemoto, Fabiano Koerich Vieira, Leonardo Moreira Sviercoski, Tanaane Ienk, Jefferson Tramontini Pagno, Viviani Gomes

**Affiliations:** ^1^Department of Internal Medicine, School of Veterinary Medicine and Animal Science, University of São Paulo, São Paulo, Brazil; ^2^Frísia Cooperative Agroindustrial, Carambeí, Paraná, Brazil

**Keywords:** assessment risk, risk analysis, biosafety, contingency plan, preventive measures

## Abstract

Biosecurity refers to a set of practices that prevents and/or controls the introduction, spread, and elimination of harmful biological agents in a production system. In this study, we aimed to survey the biosecurity practices and determine their correlation with the size of production systems. A biosecurity assessment form was provided to 69 farms in the Campos Gerais region of Paraná, Brazil. The questionnaire was divided into two sections: general and bovine viral diarrhea virus- and bovine herpesvirus type-1-specific sections. The general section covered topics on traffic control, quarantine and animal isolation, hygiene practices, carcass disposal, and disease monitoring/control. The specific section consisted of questions on the reproductive and respiratory factors, use of antimicrobials, and vaccination schedule. The 69 farms were also classified into small (≤ 61), medium (62–201), and large (≥ 202) size farms based on the number of lactating cows. Moreover, multiple correspondence analysis (MCA) was performed between the biosecurity measures and farm size. The main risk factors and variability were related to the traffic control of people, animals, and vehicles/equipment, animal quarantine/isolation, and hygiene practices. MCA revealed that the small farms exhibited a lack of biosecurity measures, including those related to traffic control, animal quarantine, and hygiene. In medium-size farms, contact between bovine animals of different ages and difficulty in animal isolation in the quarantine system were among the main risk factors. In contrast, isolation of sick animals was easy, but the need to frequently purchase cattle was an important risk factor in large farms. These results highlight the relationship between biosecurity measures and farm size, providing valuable insights for the development of better biosecurity plans for production systems.

## 1 Introduction

The Food and Agriculture Organization of the United Nations and World Health Organization describe biosecurity as an integrated approach encompassing many policy and regulatory frameworks to analyze and manage the food safety and public, animal, and plant health risks, including associated environmental risks (1). Since 2007, biosecurity is a key element of Animal Health Strategy of the European Union (2). It is also included in the preparedness plan of the European Center for Disease Prevention and Control (3) and the objectives of the International Health Regulations adopted in 2005 (4).

Biosecurity in animal production involves a set of actions to prevent or minimize the risks in production systems. The One Health approach recognizes the fundamental roles of biosecurity in regulation, animal welfare, traceability, food safety, public health, and protection against animal product trafficking (5). Integration of biosecurity measures into production systems, such as dairy farms and pig farms, is a promising strategy to reduce the use of antimicrobials (6–8).

In addition to being the largest beef exporter in 2021, Brazil has the third-largest dairy herd population and fifth-largest cattle volume worldwide (9). However, lack of national guidelines and incentives for farmers to introduce biosecurity measures has impacted the economic development and animal health in the country. Introduction of biosecurity measures is economically viable when the investment is less than the direct and indirect costs incurred by diseases and/or outbreaks in farms (10).

In this study, we aimed to survey the biosecurity practices integrated into the bovine viral diarrhea virus (BVDV) and bovine herpesvirus type-1 (BoHV-1) control program in the Campos Gerais region of Paraná (Brazil) and determine whether farm size is associated with the biosecurity practices in dairy farms. The control program consisted of three sub-projects: (i) epidemiological and economic analyses of the feasibility of the BVDV control program, (ii) evaluation of the serological response against BVDV and BoHV-1, and (iii) evaluation of the biosecurity practices in farms.

## 2 Materials and methods

### 2.1 Characterization of dairy farms

Dairy farms involved in this study were geographically distributed across nine municipalities in the region surrounding Carambei, Paraná, within a 113 km radius. In total, 69 dairy production systems using Holstein cattle were included in this study. Moreover, 1–913 lactating cows, producing an average of 31 L of milk per cow daily, were involved in this study. The average number of days that the farms produced milk was 197 d (27–300), and the somatic cell count was 60–550 cells/mL. The complete herd data, including the number of animals and type of housing in each category, are provided in Supplementary File S1.^1^

### 2.2 Biosecurity assessment

This study was conducted from January 2020 to January 2022. In 2020, a biosecurity assessment questionnaire was developed with two sections, (1) General Risk Assessment (26 questions) and (2) Risk Assessment for BVDV and BoHV-1 (36 questions), with a total of 62 questions. The first section of the questionnaire was adapted from the Center for Food Security and Public Health of the University of Iowa. The second section involved questions on the risk factors associated with BVDV and BoHV-1. Both sections included topics on control of access by people, animals, and vehicles, hygiene practices, animal quarantine and isolation, equipment monitoring and control, antimicrobial use, and vaccination.

The questionnaire was distributed by three technical veterinarians, cooperatives, and members of the research team. The team was divided into pairs to simultaneously distribute the questionnaire to different farms. On average, 35–50 min were needed to complete the questionnaire. After assessing the questionnaire, an on-site visit was conducted to ensure that the participant responses aligned with the observed conditions on the farm. This approach aimed to enhance the credibility of the collected data. English version of the 62-question questionnaire is available online (see Text footnote 1) and in Supplementary File S2.

### 2.3 Determination of the degree of biosecurity

The degree of biosecurity in the farms was determined by scoring (0–10) each question in the two sections of the questionnaire. Of the 62 questions, 50 were scored, and individual scores of the 69 respondents were determined. Notably, not every “yes” indicates an optimal biosecurity procedure to achieve the maximum score for each item evaluated (10 points). The maximum achievable score on the questionnaire was 500 (Supplementary File S3; see Text footnote 1).

Based on this score, we proposed a scale for the degree of risk assessment based on articles in scientific literature on cattle and other animal species (8, 10–12). The level of biosecurity was divided into six categories according to the percentage of best biosecurity practices adopted: very high for less than 25% (0–125 points), high for 26–50% (126–250 points), medium-high for 51–75% (251–375 points), medium for 76–80% (376–399 points), medium-low for 81–90% (400–449 points), and low for 91–100% (450–500 points). Similarly, to evaluate the degree of risk, 8 out of 10 questions (Q27, Q28, Q29, Q30, Q32, Q33, Q34, and Q39) in section two of the questionnaire were given a score of 0–10. Based on the score obtained, the degree of risk was divided into three levels: high (≥50 points), medium (40 points), and low (≤30 points). The score distribution was divided into quartiles, where the low, medium, and high levels corresponded to the Q1, Q2 (median), and Q3 quartiles, respectively (Supplementary File S4; see Text footnote 1).

### 2.4 Statistical analyses

Statistical analyses were conducted using the Statistical Analysis System (SAS) software v.9.4 (SAS Institute Inc., NC, USA). All qualitative nominal variables were transformed into binary answers (yes/no) to verify the association between farm size and biosecurity practices. The farms were divided according to the farm size, which was determined by the number of lactating cows in the herd. The farms were classified as small (1–61), medium-size (62–201), and large (202–1913 lactating cows) farms by dividing them into quartiles.

Multiple correspondence analysis (MCA) was conducted to identify the association between biosecurity practices and farm size using the JMP program as part of SAS (version 17, premium). MCA creates dimensions using the variance of all observed variables to represent the latent variables, that is, variables that cannot be directly observed or measured but are inferred from other related variables. The dimensions are then presented in a descending order of the amount of variation (dimensions 1, 2, 3, … n), enabling the identification of variables contributing the most to their creation (13).

## 3 Results

### 3.1 General biosecurity measures

Answers to the questionnaire are presented in Supplementary File S5 (see Text footnote 1).

#### 3.1.1 People movement

The farms had 1–70 visitors per week; on average, the farms had seven weekly visitors. However, only 15% of the farms, mainly large farms, had implemented a visitor policy. In 14% of the farms, employees were in contact with cattle outside the farms.

#### 3.1.2 Animal movement

Introduction and removal of the same animals from the farms for various events, reproductive management, off-farm rearing, or external veterinary care were observed in 41% of the farms (28/67), with 19% in medium-size, 10% in large, and 8% in small farms. New animals were acquired annually, monthly, or weekly, particularly from medium-size and large farms. Moreover, 7% (5/69) of the farms included pregnant cows. In approximately a quarter of the farms, cattle were in contact with other animals of the same species but of different ages (27%, 19/69), mainly heifers under 12 months of age (7/19), weaned calves (4/19), and dry cows (4/19). In addition to the cattle, the following animal species lived on the farms: dogs, cats, birds, chicken, geese, horses, goats, sheep, and some wild animals, such as curassows, agoutis, coatis, howler monkeys, guinea fowls, birds, deer, raccoons, capybara, lizards, wild boars, jaguars, and rats.

#### 3.1.3 Vehicles and equipment

No specific area for vehicle parking was available in small farms, which were typically close to healthy animal facilities. Most small farms did not have trucks to transport the cattle for their use (81%); they rented trucks for activities, such as sending animals to events, reproductive management, off-farm rearing, external veterinary care, and slaughter. In addition, due to the proximity of farms in the study region, the same truck was often used to transport several animals from different farms. Vehicles used to dispose the animals (sick or male calves) also entered the farms, particularly medium-size farms.

#### 3.1.4 Sanitary measures

Among the hygiene practices evaluated in this study, cleaning and disinfection processes (CDPs; 84%, 58/69) were commonly performed in calf stalls/cages slated to receive neonates. Most farmers reported using machinery and/or equipment with a dual function, including handling (feeding) and waste disposal, regardless of farm size. Of these, only 3% (2/54) of the equipment was cleaned and disinfected before being used again to distribute the food to the animals on medium-size and large farms. In addition, 53% (37/69) of the farmers reported washing their hands with soap and water before handling the animals. No vehicle wheel disinfection system or disinfection arch was available on the farms for cleaning and disinfecting the vehicles, trucks, or cars entering the farms. Furthermore, in case of abortions in the farms, CDPs were not performed in almost all farms (85%, 59/69).

#### 3.1.5 Quarantine and isolation

Small- and medium-size farms reported difficulty in isolating animals on the farm (59%). The isolation and quarantine areas were usually close to the facilities for healthy animals in the herd, especially on small farms. The quarantine period reported by the producers was 0–60 days. When introducing new animals into the herd, only 20 farms tested for diseases, with half of them from small farms, nine from medium-size farms, and one from a large farm. Tests were performed for brucellosis (19 citations), tuberculosis (19 citations), and BVD (one citation).

#### 3.1.6 Preventive measures and farm management

Necropsies were not performed in 59% (41/69) of the farms with an unknown cause of death, mainly in small- and medium-size farms. Most carcasses were buried away from the property and allowed to decompose naturally, with a few animals left in ditches or out in the open. The treatment of manure and/or bedding was a common practice, given the cooperative’s encouragement, with 46% of both being carried out, 40% only for manure, and 12% for bedding. The main waste treatments cited are manure pits, cesspits, and biodigesters. Animal bedding was subjected to lime application and only one farm sprayed creolin and another farm applied potassium monopersulfate (Virkon). Finally, we have determined the most frequently utilized classes of antimicrobials for treating respiratory illnesses on farms. Our findings reveal a total of 8 classes, including aminoglycosides, amphenicols, beta-lactams, macrolides, quinolones, and tetracyclines. Overall, quinolones and macrolides are the primary choices for small and medium-sized farms, while amphenicols and macrolides are the preferred options for larger farms.

### 3.2 Biosecurity measures to control BVDV and BoHV-1

#### 3.2.1 Assessment of risk perception

Determination of the degree of risk perception revealed that the majority of producers on small, medium-size, and large farms had a high degree of risk perception (83%, 15/18; 94%, 32/34; 88%, 15/17, respectively). In general, producers knew about the occurrence of BVDV and BoHV-1 viruses in their farms and sought information on measures to protect themselves. Moreover, most of the diseases cited by the producers were tuberculosis (43 times), brucellosis (37 times), BVD (24 times), mastitis (15 times), and Infectious Bovine Rhinotracheitis (14 times).

#### 3.2.2 Reproductive practices

Artificial insemination was the only reproductive biotechnology used in these systems. Follicular Aspiration for *In Vitro* Fertilization and embryo transfer was not performed. In the case of bulls, three medium-size farms and two large farms requested tests on the efficiency and reproductive health of males, and four farms did not answer this question. The main causes of culling in cows are non-pregnancy, mammary gland disorders, uterine disorders, such as metritis, other reproductive causes, and low milk production.

The frequency of abortions in the herd, in terms of absolute numbers, was higher when the frequency of abortions was annual, especially in small- and medium-size farms. As the frequency of abortions increased, the number of farms decreased, regardless of size. In addition, females were generally kept together with other animals (79%; 55/69) without any tests being carried out to identify possible agents causing reproductive disorders.

#### 3.2.3 Degree of risk assessment

**Figure 1:**
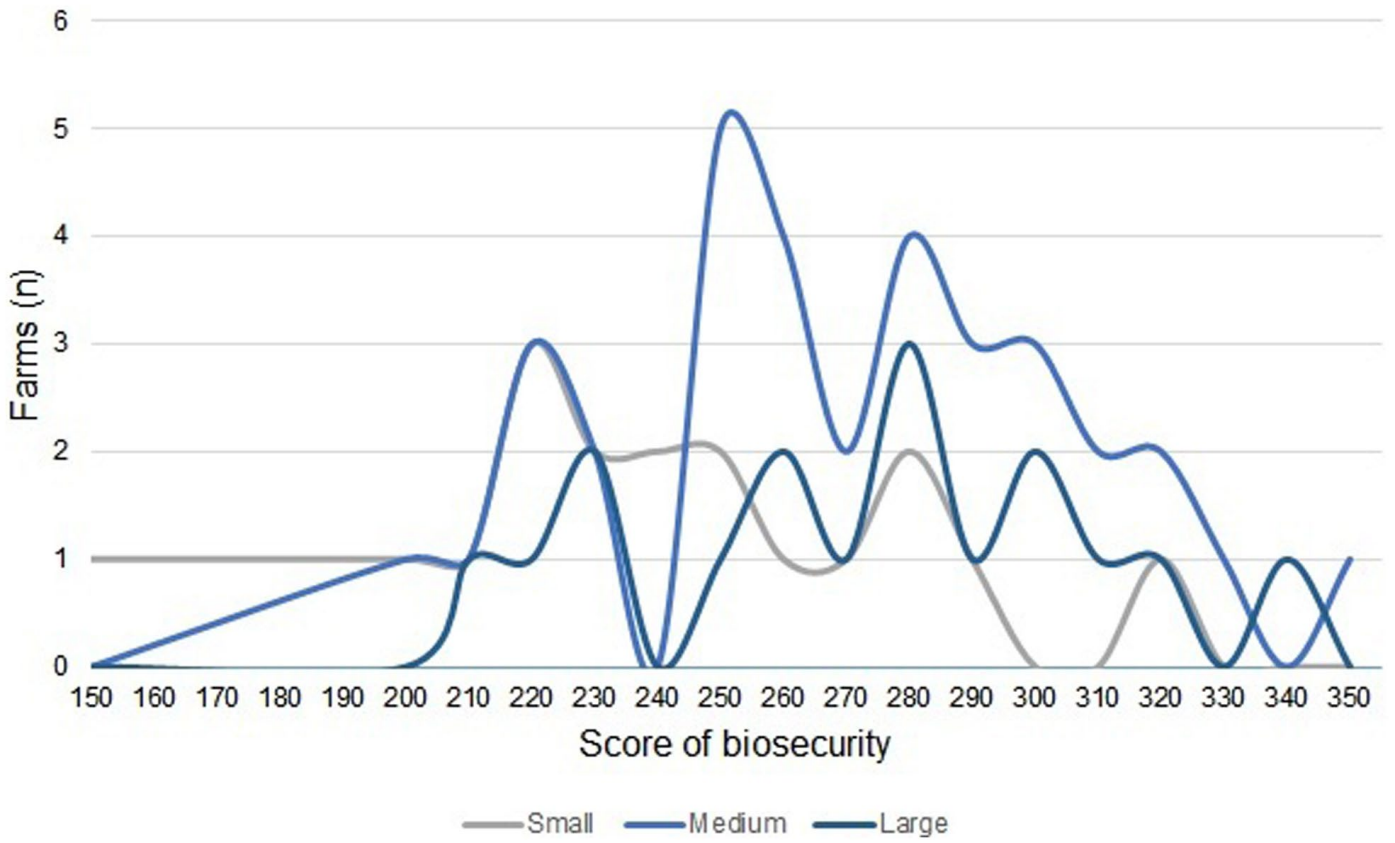
Distribution of farms and biosecurity score by farm size.

Here, average risk score of the farms was 263 points. According to the scale used to determine the degree of biosecurity, the farms presented medium-to-high risk. The scores of the farms were in the range of 150–350, falling in the high- and medium-high risk levels (Figure 1).

Degree of risk assessment revealed scores of 150–320, 200–350, and 210–340 points for small, medium-size, and large farms.

### 3.3 MCA of the farm size and general and specific biosecurity practices for BVDV and BoHV-1

MCA of the biosecurity data and size explained 100% of the variation between the 69 farms and observed variables. To better visualize and understand the data, we grouped the variables according to their corresponding topics (traffic control of people and vehicles, animal traffic control, animal quarantine, hygiene practices, reproductive management, and calving). In all analyses, the MCA generated two dimensions (d1 – x-axis and d2 – y-axis), varying in terms of their contribution (%) to each dimension according to the measures analyzed.

**Figure 2:**
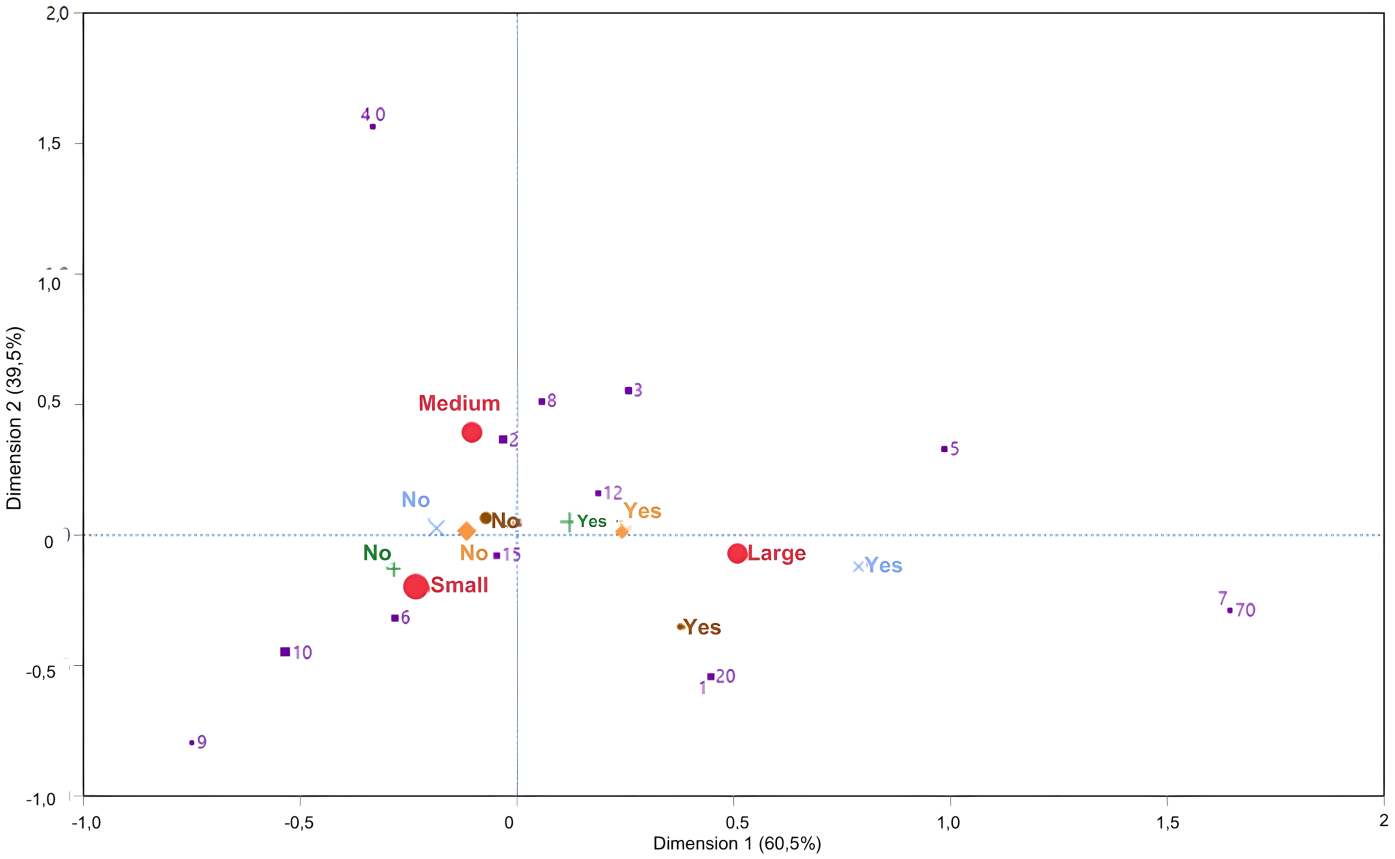
Multiple correspondence analysis to evaluate the association of biosecurity measures aimed at controlling traffic of people and vehicles\equipment according to the size of the properties. Large red circle – size of the farms; Small brown circle – presence of protocol for visitors (visitors’ diary, minimizing contact With the animals, use of clothing provided by the farm itself such as overalls and spare boots, etc.); Purple square – Number of visitors (vets, milk trucks, food deliveries, etc.) entering the property each week; Blue cross – presence of tractors or trucks for towing and/or transporting livestock (except waste animals); Green cross – Does the truck or vehicle for transporting waste animals (sick animals and male calves) enter the property; Orange diamond – Is there a specific area for parking vehicles on the property?

In the first analysis, small farms and their associated practices were located in the lower-left quadrant of the MCA graph, medium-size farms were in the upper-left quadrant, and large farms were in the lower-right quadrant (d1: 60% and d2: 39%). In general, small farms lacked biosecurity measures associated with prohibiting the entry of vehicles for transporting disposed animals into clean areas of the property. Larger farms were associated with the presence of biosecurity measures to control human and vehicle traffic, except for vehicles entering clean areas to dispose of animals (Figure 2).

**Figure 3:**
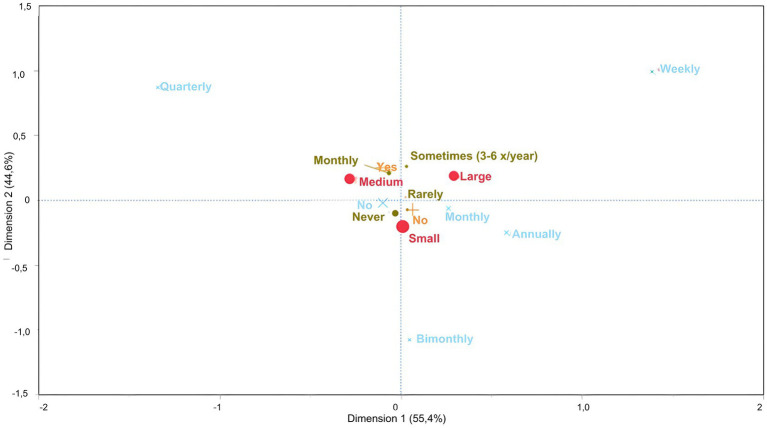
Multiple correspondence analysis to evaluate the association between animal traffic control measures and the size of dairy farms classified as small, medium and large, according to the number of lactating cows. Large circle – farm size; Small green circle – how often do animals leave and rejoin the herd (shows, embryo transfer, external clinical care with a veterinarian. Exhibitions, etc.); Orange cross – Do the animals on the property have contact with other animals of the same species of different ages; Blue cross – How often are new animals introduced into the herd?

In the second analysis, the variability obtained from the first dimension accounted for 55% (x-axis) and the second accounted for 44% (y-axis). Small farms were located in the lower-medial portion, medium-size farms remained in the same quadrant as in the previous analysis, and large farms were in the upper right quadrant. On small farms, we found that the animals did not leave the property or rarely left; there was no introduction of new animals or contact between animals of different ages. On medium-size properties, cattle go out monthly for external events, and adult cattle have contact with individuals of other ages. However, on large properties, the animals went out three to six times a year for external events (Figure 3).

**Figure 4:**
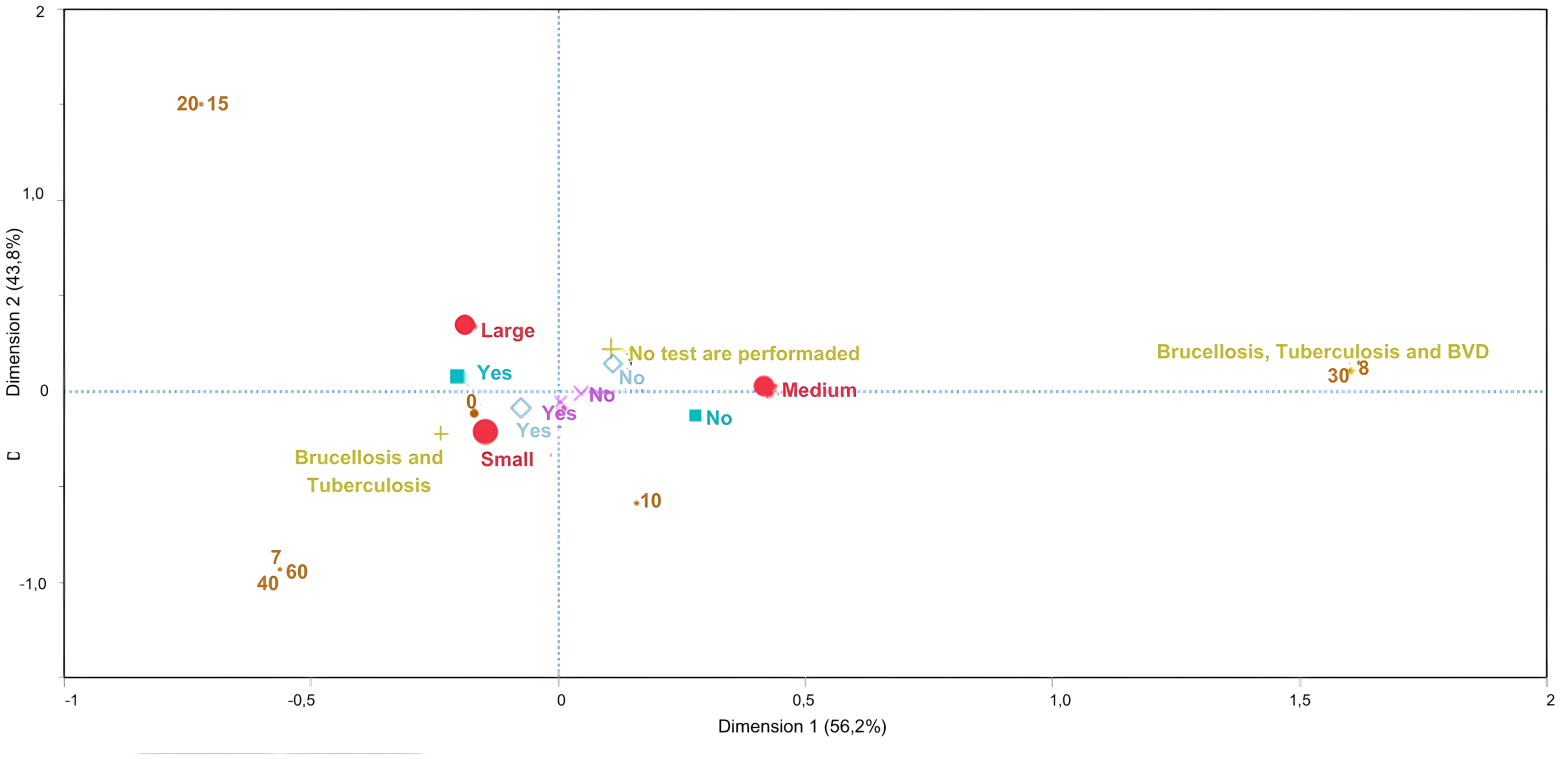
Multiple correspondence analysis to the assess association between biosecurity practices for quarantine/isolation and the size of dairy farms classified as small, medium and large, according to the number of lactating cows. Large red circle – size of farms; Small brown circle – How long are animals kept in quarantine (days)?; Greenish cross – What test is carried out before introducing an animal?; Lilac cross – Are you able to easily isolate animals in quarantine? Light blue square – If there is a sick animal, can you easily isolate it from the other healthy animals in the herd? Medium blue rhombus – Is the isolation or quarantine facility for sick animals close to the facilities for healthy animals?

Among the measures related to the quarantine and isolation system, the MCA revealed that small farms did not quarantine their animals, although they conducted tests for Brucellosis and Tuberculosis before introducing a new animal. On these farms, the locations where sick animals were isolated were close to the facilities for healthy individuals. On medium-size farms, it was difficult to isolate sick animals from farms. On the other hand, on large farms, there were no difficulties, and sick animals were isolated. In this case, dimension 1 accounted for 56% of the variability and dimension 2 accounted for 43% (Figure 4).

**Figure 5:**
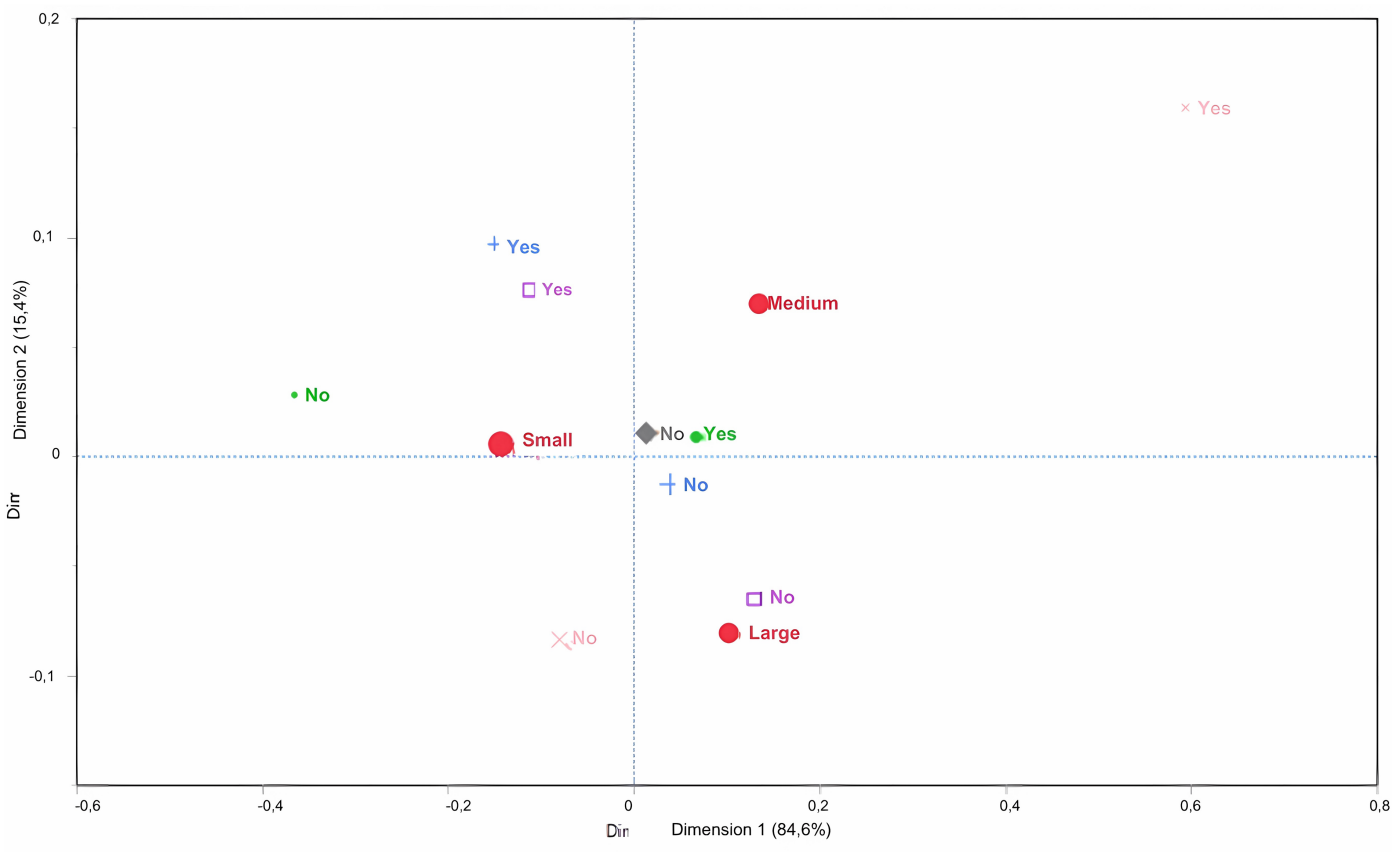
Multiple correspondence analysis to assess the association between biosecurity practices for hygiene and the size of dairy farms (classified as small, medium and large), according to the number of lactating cows. Large red circle – Size of farm; Small green circle – DO you clean and disinfect the calf cages for receiving newborn calves? Blue cross – DO you use different equipment for feeding and handling waste? Light pink cross – DO you do clean and disinfect equipment with dual functions? Purple square – Is everyone required to wash their hands with soap and water before handling the animals? Gray rhombus – Is there a wheelchair at the entrance to the property?

With regard to hygiene measures (d1: 84% and d2: 15.4%), there was a simple relationship between small farms and not carrying out cleaning and disinfection processes of cages and stalls in the calf yard, the use of different equipment for feeding and waste management, and the act of washing hands before handling animals. Medium-size properties showed no direct correspondence, whereas large properties did not require hand hygiene before handling the animals (Figure 5).

**Figure 6:**
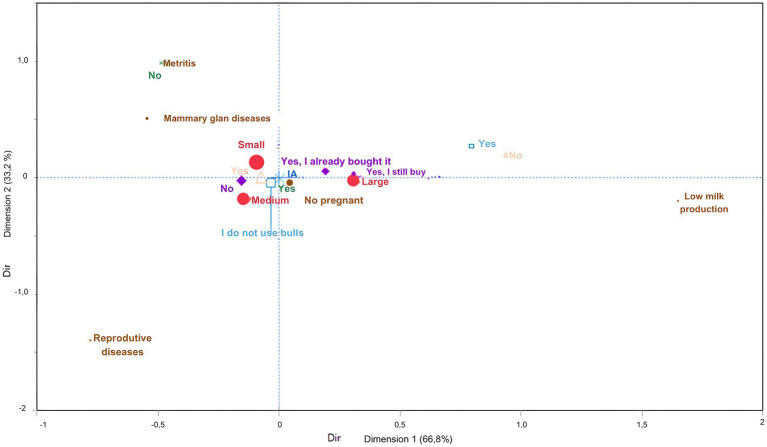
Multiple correspondence analysis to the assess association between productive management practices and dairy farm size. Large red circle – Farm size; Small brown circle – What is the main reason for cows being discarded? Blue cross – How are the females in the herd reproduced? Dark green cross – If artificial insemination (Al) is used, is it done by properly trained staff, using clean techniques and instruments? Light blue square – In the case of own bulls, do you require tests on their reproductive efficiency and health? Purple diamond – Have you ever bought/are you still buying pregnant animals’? Light pink triangle – Are reproductive disorders in animals recorded in notebooks or computers (e.g., calving difficulties, metritis, retained placenta, etc.)?

Heifers and cows did not need calving assistance on small farms, and the average age of heifers suitable for breeding was 13 months. Calving assistance was needed in medium-size and large farms and similarly associated with both. Major reasons for culling cows on small farms were metritis and mammary gland disorders; however, no such conditions were observed in medium-size farms. On large farms, the primary reason for culling cows was failure to become pregnant. Small- and medium-size farms did not purchase pregnant cows. In contrast, large farms bought pregnant cows (d1: 67% and d2: 33%; Figure 6). Reproductive disorders were only observed in small- and medium-size farms.

**Figure 7:**
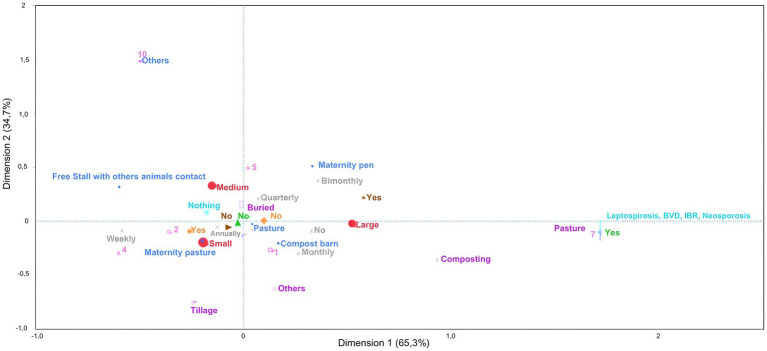
Multiple correspondence analysis to assess the association between reproductive factors related to childbirth and the size of dairy farms classified as small, medium and large. Large red circle – Size of the farms; Small blue circle – Where do the females give birth? Brown arrowhead on the right – Is the place disinfected after the females abort? Gray cross – What is the occurrence Of females aborting on the farm? Pink square – Number Of abortions; Orange rhombus – Is the aborted female separated from the herd? Green triangle – In relation to the aborted fetus, is any biological material from the animal sent to the laboratory? Turquoise asterisk – What test(s) is (are) requested When the material from the aborted fetus is sent? Lilac perpendicular rectangle – What is the destination of the aborted calf/fetus?

Analysis of biosecurity measures related to calving (d1: 65% and d2: 34%) revealed that small farms were associated with the separation of females with miscarriages, who were housed in maternity paddocks, annual occurrence of miscarriages, and failure to disinfect the site afterward. In addition, the aborted material was not collected or sent for analysis or pathogen testing. Medium-size farms did not correspond to any biosecurity practices related to this topic. Large farms were associated with cleaning and disinfecting the site after the event and absence or monthly frequency of these episodes. In the same quadrant, females in compost barns were associated with a monthly occurrence, whereas those in maternity stalls were associated with a bimonthly occurrence of abortions (Figure 7).

## 4 Discussion

This study characterized the general and specific biosecurity measures for BVDV and BoHV-1 and demonstrated their association with the size of dairy farms. The answers, mainly those on the access control for people, animals, and vehicles, animal quarantine and isolation, and hygiene practices, varied considerably among the respondents.

In general, biosecurity measures have been poorly adopted in dairy farms. Small dairy farms had the lowest degree of biosecurity, as indicated by the biosecurity scores. This trend is also observed in dairy, mixed (dairy and beef), and small ruminant farms (14). One study reported the increase in biosecurity with the farm size (15).

Analysis of the biosecurity practices to control human traffic revealed a wide variation in the number of weekly visitors to farms. Only 15% of farms had visitor policies in place, of which only 4% were small- and medium-size farms. The scarcity of appropriate biosecurity measures has also been reported in other farms (16–18) and large beef cattle farms (>4,000 farms) (19).

The lack of designated vehicle-parking areas near cattle facilities was more common on small farms, whereas the entry of trucks carrying animals for slaughter or disposal was more prevalent on medium-size farms. Brand et al. (19) also reported this practice in beef cattle farms with more than thousand heads. Most of the systems did not have trucks or vehicles for towing/transporting the animals, and they were mostly on large farms, which is consistent with other studies (20). Financial resources can be an important factor for small and medium-size farmers to adopt certain biosecurity measures (21). Simple and cost-effective biosecurity measures can be readily implemented, including communicating access restrictions to visitors, enforcing proper handling of animals, and ensuring that visitors sanitize their hands, boots, and shoes before entering free stalls or bovine facilities. Additionally, having a designated parking area for vehicles is essential and should be emphasized.

The practice of purchasing animals was mainly found in medium and large farms; medium (61%) and large (75%) farms bought more animals than small farms (40%) (22). The purchase of animals (replacement heifers) from small- and medium-size farms is a risk factor for the introduction and spread of BVDV (23). Farms with more cattle implement significantly fewer bioexclusion measures, despite the greater impact of an infectious disease being introduced into the herd (24). Furthermore, in dairy farms, the proportion of heifers reared onsite decreases as the herd size increases (25). Therefore, it is important for farms to take measures to prevent the introduction of pathogens into farms. One such measure is quarantining animals before reintroducing them to the herd. Diagnostic tests should be performed during the quarantine. Additionally, when transporting animals, it is important to avoid mixing batches from different farms and to sanitize the interior and exterior of the transport vehicles.

Regarding hygiene practices, sharing of machinery for handling waste and feeding animals was found in most cases, regardless of the size of the production system. The practice of CDP of stalls/cages in calves and hand washing before handling animals was widely observed in medium-size systems, corroborating what was described for medium and large farms (14). It is worth highlighting, specifically in this section of the questionnaire, the possible inconsistencies in which the respondents were not honest in their answers. CDPs are essential to prevent the spread of pathogens. To achieve this, it is necessary to clean and disinfect all vehicles that regularly enter a property. Additionally, daily cleaning and disinfection of trucks shared for transporting animals are crucial. These measures may require significant financial investment or employee labor to implement them.

Small- and medium-size farms have found it difficult to isolate farm animals using quarantine systems. Considering the need to avoid direct and indirect contact with other animals, the infrastructure and physical space of these properties may have been obstacles to implementing this measure. Sahlström et al. (20) found that this measure was more widely adopted in beef farms than in dairy farms, and was also associated with the size of the production systems. According to Hoe and Ruegg (22), larger farms isolate sick animals more easily and carry out more diagnostic tests or examinations when new animals are acquired compared to smaller systems. According to the World Organization for Animal Health (4), the quarantine site must be under the supervision of a veterinary authority, the animals must be kept in isolation without direct or indirect contact with other animals for a specific period, and tests and treatments of these animals must be carried out as necessary. To manage and prevent diseases, farms must be able to identify agents that could potentially be brought in and those that are already present on the property. Brazil recommends a quarantine and observation period of at least seven days, along with diagnostic tests for brucellosis, tuberculosis, and reproductive and respiratory diseases in production systems (26).

Here, frequencies of each BVD risk perception group were similar (high: 37%, medium: 31%, and low: 30%). Although no significant differences were observed, the knowledge of biosecurity measures was low among livestock farmers, but their perception of their importance, interest in seeking information, and adoption of such measures were good (27).

The sparing and judicious use of antimicrobials is essential for effective treatment of infections. Due to the limited alternatives to antimicrobials, biosecurity measures are necessary to ensure their appropriate use. This study found that the most commonly used antimicrobial classes for treating respiratory diseases in small- and medium-size farms were quinolones, followed by macrolides, whereas large farms preferred amphenicols and macrolides. The European Medicines Agency has classified available antimicrobials into four categories (A-Avoid, B-Restrict, C-Caution, and D-Prudent) to guide veterinarians on the use of antimicrobials and their impact on human and food health (28). The three classes identified in this study fall under category B (Restricted use; quinolones) and C (use with caution; amphenicols and macrolides). Farm veterinarians should be provided access to this information and choose class D antimicrobials to prevent antimicrobial resistance. Large farms tend to use more antimicrobials. Some measures to reduce their use on dairy farms include (i) cleaning and disinfecting facilities, (ii) using replaceable bedding materials, (iii) avoiding contact with other farms, (iv) proper quarantine when introducing new animals, and (v) proper management of mammary gland health (29).

Different MCA analyses showed that farms can be classified into three categories based on the implementation of biosecurity measures: those with the most biosecurity measures, those with few measures, and those with no measures. Generally, small farms do not have any biosecurity practices in place or avoid certain risk factors, owing to their small size. Medium-size farms are in transition with only a few biosecurity measures. Large farms have the most biosecurity measures, but they also face intrinsic risk factors owing to the expansion of their farms. When we consider the risk score obtained, we realize how few biosecurity measures are adopted in general, as the risk levels obtained were high and medium-high.

Successful implementation of the recommended management practices depends on the risk perception of the farmers, including the risk they are willing to take and the associated consequences, and the importance attached to a particular biosecurity measure (27). Therefore, the perceived effectiveness of the recommended guidelines, feasibility, and technical knowledge of the subject increase the likelihood of adopting the biosecurity measures in production systems. Here, biosecurity measures recommended for different study groups are described in a biosecurity manual available online at the Open Book Portal of USP.^2^

Here, our findings indicate an effective approach to improve the management of BVDV and BoHV-1 in Brazilian farms. Our findings suggest regular diagnostic tests and introduction of biosecurity measures in dairy farms. Notably, no state or federal control or eradication initiatives have been proposed in Brazil. Therefore, our findings can serve as a valuable resource for public and private organizations aiming to establish effective control programs and offer guidance to cattle farmers regarding disease control.

## 5 Conclusion

Biosecurity measures are essential in animal production systems. Currently, these measures are poorly implemented in various systems. Here, our findings revealed the association between farm size and biosecurity measures, highlighting the importance of the assertive implementation of preventive measures in production systems.

## Data availability statement

The datasets generated for this study can be found online via the following link: https://doi.org/10.5281/zenodo.10027096.

## Ethics statement

The studies involving humans were approved by Platform Brazil (number: 37108020.2.1001.5390). The studies were conducted in accordance with the local legislation and institutional requirements. The participants provided their written informed consent to participate in this study. The animal studies were approved by Ethics Committee on Animal Use of the Faculty of Veterinary Medicine and Animal Science of the University of São Paulo (CEUA/FMVZ) approved this research (protocol number: 8776020221). The studies were conducted in accordance with the local legislation and institutional requirements. Written informed consent was obtained from the owners for the participation of their animals in this study.

## Author contributions

JF: Data curation, Formal analysis, Investigation, Writing – original draft, Writing – review & editing. CB: Investigation, Project administration, Supervision, Validation, Writing – original draft. BN: Writing – review & editing. FV: Funding acquisition, Investigation, Project administration, Supervision, Writing – original draft. LS: Investigation, Writing – original draft. TI: Investigation, Writing – original draft. JP: Funding acquisition, Project administration, Resources, Supervision, Writing – original draft. VG: Funding acquisition, Investigation, Methodology, Project administration, Resources, Supervision, Writing – review & editing.

## Footnotes

^1^ https://doi.org/10.5281/zenodo.10620692

^2^ https://doi.org/10.11606/9786587778068
