# Supplementary material for: Biosecurity practices in the dairy farms of southern Brazil
Source: Front Vet Sci. 2024 Mar 27;11:1326688. doi: 10.3389/fvets.2024.1326688 (PMC11004291; doi:10.3389/fvets.2024.1326688)
Supplement: Supplementary file 2 [file Table_1.DOCX]

S1. Table 1 - Distribution of animal categories and type of housing in dairy production systems in the Campos Gerais region of Parana.

| **Variables** | **Animal categories of dairy farm** | | | | | | |
| --- | --- | --- | --- | --- | --- | --- | --- |
|  | Pre-weaned calves | Weaned calves | Heifers  (9-12mo) | Heifers  (13-24mo) | Dry cows  (60-30 days pre-partum) | Dry cows  (Below 29 days pre-partum) | Lactating cows |
| Farms (n) | 67² | 66² | 55³ | 59³ | 64² | 66² | 68² |
| Average | 27 | 48 | 35 | 70 | 17 | 17 | 183 |
| Median | 15 | 20 | 20 | 42 | 10 | 9 | 97 |
| Min¹ | 1 | 0 | 0 | 0 | 1 | 1 | 16 |
| Max¹ | 250 | 600 | 170 | 500 | 170 | 129 | 1913 |
| *Kind of housing* | | | | | | | |
| Individual Calf Housing | 70.14%  (47/67) | 3.03%  (2/66) | - | - | - | - | - |
| Collective cage | - | 33.33%  (22/66) | 1.81%  (1/55) | - | - | - | - |
| Tropical Calf Housing | 14.92%  (10/67) | - | - | - | - | - | - |
| Argentine Calf Housing | 1.49%  (1/67) | - | - | - | - | - | - |
| Cage Free | 10.44%  (7/67) | - | - | - | - | - | - |
| Calf Feeder | 2.98%  (2/67) | - | - | - | - | - | - |
| Pasture | - | 59.09%  (39/66) | 90.90%  (50/55) | 89.83%  (53/59) | 73.43%  (47/64) | 66.67%  (44/66) | 42.64%  (29/68) |
| Free Stall | - | 1.51%  (1/66) | 3.63%  (2/55) | 5.08%  (3/59) | 14.06%  (9/64) | 16.66%  (11/66) | 44.11%  (30/68) |
| Compost Barn | - | 3.03%  (2/66) | 1.81%  (1/55) | 3.38%  (2/59) | 7.81%  (5/64) | 12.12%  (8/66) | 11.76%  (8/68) |
| Free Stall with pasture access | - | - | 1.81%  (1/55) | 1.69%  (1/59) | 4.68%  (3/64) | 4.54%  (3/66) | 1.47%  (1/68) |
| Other | 2.98%  (2/67) | - | - | - | - | - | - |

Legend: ¹Min = minimum; Max – maximum. ² Small sample size due to blank answers on the questionnaire. ³ 14 farmers were rearing heifers from 9 to 12 months of age outdoors; 10 farmers did outdoor rearing from 13-24 months of age.
