# Supplementary material for: Biosecurity practices in the dairy farms of southern Brazil
Source: Front Vet Sci. 2024 Mar 27;11:1326688. doi: 10.3389/fvets.2024.1326688 (PMC11004291; doi:10.3389/fvets.2024.1326688)
Supplement: Supplementary file 3 [file Table_2.DOCX]

Biosecurity assessment questionnaire for dairy herds

| Farms’ Name: | Date: |
| --- | --- |
| Address: | |
| Phone: | |
| Owner/manager: | E-mail: |
| Reviewed by: | |

**PART I - Questionnaires for general assessment (questions 1 to 26).**

| 1. Is cleaning and disinfection of calf cages/bays for receiving newborn calves carried out? | ( ) Yes ( ) No |
| --- | --- |
| 2. How many visitors (vets, milk trucks, food deliveries, etc.) enter the farm each week? |  |
| 3. Do you have any protocol for visitors (visitor diary, minimizing contact with animals, use of clothing provided by the farm itself such as overalls and spare boots)  **If yes,** please describe. | ( ) Yes ( ) No  Describe: |
| 4. Do you ask your employees to avoid contact with cattle outside your property? | ( ) Yes ( ) No |
| 5. Are there requirements that everyone always washes their hands with soap and water before handling their animals? | ( ) Yes ( ) No |
| 6. How often animals leave and re-enter the herd (shows, embryo transfer, external clinical care with veterinarian, exhibitions, etc.): | ( ) Never  ( ) Rarely (1-2 times a year)  ( ) Sometimes (3-6 times a year)  ( ) Frequently (monthly)  ( ) Very often (fortnightly/weekly) |
| 7. Do the animals on the farm have contact with other animals of the same species of different ages?  If **yes,** with which category? | ( ) Yes ( ) No  Choose the animal category:  ( ) Calves  ( ) Weaned calves  ( ) Heifers  ( ) Dry cows  ( ) Lactating cows  ( ) Newly calved cows |
| 8. Do you have tractors or trucks for towing and/or transporting livestock (except disposal of animals)?  **If yes**, what is the method of disinfection? | ( ) Yes  ( ) No  Disinfection method: _______________________________ |
| 9. Does the truck or vehicle for the transportation of disposal animals (sick and male calves) enter the property?  **If yes**, what is the method of cleaning and disinfecting the vehicle at the farm entrance? | ( ) Yes  ( ) No  Disinfection method: _______________________________ |
| 10. Is there a specific parking area for vehicles on the property? | ( ) Yes ( ) No |
| 11. Is the general vehicle parking area far from the main animal housing areas? | ( ) Yes ( ) No |
| 12. Is there a vehicle wheel disinfection system at the entrance to the property? | ( ) Yes ( ) No |
| 13. How often are new animals introduced into the herd? | ( ) Does not occur  ( ) Annually  ( ) Monthly  ( ) Weekly |
| 14. Do you have a facility for isolating animals in a quarantine system? If **yes,** how long are the animals kept on average?  Are any tests carried out on the animals before introducing them into the herd? **If yes**, which one? | ( ) Yes ( ) No  Isolation Time: ________________________  List tests: ________________________ |
| 15. If there are any sick animals, can they be easily isolated from the other healthy animals in the herd? | ( ) Yes ( ) No |
| 16. Is the isolation or quarantine place for sick animals close to the premises of resident (healthy) animals? | ( ) Yes ( ) No |
| 17. List other domestic animals residing on the farm |  |
| 18. Do you use different equipment for feeding and manure handling?  If **not,** do you do equipment disinfected after the end of each operation? | ( ) Yes ( ) No  ( ) Yes  ( ) No |
| 19. Is there treatment of animal waste and/or bedding? Please mention the treatment of litter and/or manure. | ( ) Yes, both  ( ) Yes, only from waste  ( ) Yes, only from bed  ( ) No  Name the treatment: |
| 20. Do you do necropsy of animal dies unexpectedly?  If **yes**, please specify if there is a specific place for necropsy and who is responsible for performing this procedure. | ( ) Yes ( ) No  Local: ________________________ |
| 21. Where is the carcass of animals that die on the farm disposed of? | ( ) In farming  ( ) Used in composting system  ( ) The animal is buried  ( ) The carcass is incinerated  ( ) Other |
| 22. Do you use vaccines and medicines according to the specifications provided in the product package insert by the manufacturers? | ( ) Yes ( ) No |
| 23. Is there a record of the temperature monitoring of the refrigerator where vaccines and medicines that need refrigeration are stored? | ( ) Yes ( ) No |
| 24. Do you store vaccines and medicines in refrigerator to specifically for this purpose? Or does it have other purposes?  Please tick more than one alternative where applicable. | ( ) Yes ( ) No |
| 25. What is the "age" of the refrigerator where vaccines and medicines that need refrigeration are stored? |  |
| 26. List the diseases that you would not like to occur in the animals of your herd. |  |

**PART II - Specific questionnaires for perception and biosecurity assessment for BVDV and BoHV-1 (questions 27 to 62)**

| 27. Do you know what BVDV is? | ( ) Yes ( ) No |
| --- | --- |
| 28. Do you know what IBR is? | ( ) Yes ( ) No |
| 29. Do you consider your farm protected against BVDV and IBR virus? | ( ) Yes ( ) No |
| 30. Is there concern about the occurrence of these diseases in the herd? | ( ) Yes ( ) No |
| 31. Which category do you believe is more predisposed to BVDV and IBR virus?  Select more than one alternative if necessary. | ( ) Calves  ( ) Weaned calves  ( ) Heifers  ( ) Dry cows  ( ) Lactating cows  ( ) Newly calved cows  ( ) Don't know |
| 32. Have you been informed or sought information on what to do to protect your herd from BVDV and IBR virus? | ( ) Yes  ( ) I have not looked but would like more information  ( ) I am not interested |
| 33. Do you know what to do if BVDV and IBR virus affect an animal? | ( ) Yes ( ) No |
| 34. Do you think you could take measures to prevent the entry and spread of these diseases on your farm? | ( ) Yes ( ) No |
| 35. At what age is the heifer suitable for breeding on the farm? |  |
| 36. How do you is pregnancy diagnosed in your herd? (Check all that apply) | ( ) Manual palpation  ( ) Ultrasound  ( ) Activity monitors  ( ) No return to heat  ( ) Blood test  ( ) Milk test (presence of GAP)  ( ) None  ( ) Other:_____________ |
| 37. Do the heifers in your herd normally need assistance during calving?  If yes, how many on average? | ( ) Yes ( ) No  How many? ____________________ |
| 38. Do the cows in your herd generally need assistance during calving?  If yes, how many on average? | ( ) Yes ( ) No  How many? ____________________ |
| 39. Do you consider abortion a problem in your herd? | ( ) Yes ( ) No |
| 40. Where do females give birth to their calves? | ( ) Maternity stall  ( ) Maternity picket  ( ) Free stall in contact with other animals  ( ) Pasture  ( ) Other. Please specify: ______________________________ |
| 41. Do you realize site disinfection carried out after females abort? | ( ) Yes ( ) No |
| 42. When an abortion occurs, do you perform any tests on the animal? | ( ) Yes ( ) No |
| 43. Is the aborted female separated or kept from the herd? If yes, please specify. | ( ) Yes, it is separate. Please specify: ___________________________  ( ) No |
| 44. In relation to the aborted fetus, is any biological material from the animal sent to the laboratory? | ( ) Yes ( ) No |
| 45. Which test(s) is requested when sending the material of the aborted fetus? |  |
| 46. What is the fate of the aborted calf/fetus? | ( ) Farming  ( ) Used in composting system  ( ) The animal is buried  ( ) The carcass is incinerated  ( ) Other |
| 47. What is the occurrence of females aborting on your farm?  If **yes**, how many? | ( ) Weekly  ( ) Monthly  ( ) Bimonthly  ( ) Quarterly  ( ) Annual  ( ) Does not occur  Quantity: |
| 48. What is the main reproductive reason for cows being culled? | ( ) Open/ Not pregnant  ( ) Abortions  ( ) Dystocias  ( ) Metritis  ( ) Other |
| 49. How does the reproduction of females in the herd? | ( ) Herd's own breeding bulls  ( ) Third party breeding bulls  ( ) Artificial Insemination  ( ) Embryo transfer (ET; IVF)  ( ) Other |
| 50. When you used artificial insemination (AI), is it carried out by properly trained personnel, using clean techniques and instruments? | ( ) Yes ( ) No |
| 51. In the case of own bulls, are examinations of their reproductive efficiency and health required?  If yes, which tests? | ( ) Yes ( ) No  Name the examinations:  ___________________________ |
| 52. Have you ever bought or still buy pregnant animals?  If **yes**, is information provided on the animal's health, history, vaccinations, etc.? Cite. | ( ) Yes, already bought  ( ) Yes, I still buy  ( ) No  Cite:  ___________________________ |
| 53. Are reproductive disorders of the animals (calving difficulties, metritis, retained placenta, etc.) recorded in notebooks or computers? | ( ) Yes ( ) No |
| 54. Which category in your perception faces the greatest challenge for respiratory diseases? | ( ) Calves  ( ) Weaned calves  ( ) Heifers  ( ) Dry cows  ( ) Lactating cows  ( ) Newly calved cows |
| 55. Do you record the occurrence of respiratory diseases? | ( ) Yes ( ) No |
| 56. Do you have pre-established treatment protocols for respiratory diseases?  If **yes**, which one? | ( ) Yes ( ) No  Protocol: |
| 57. What is the trade name of the antimicrobials used and the duration of treatment for respiratory disease? |  |
| 58. Do you use antimicrobials preventively in healthy farm animals? | ( ) Yes ( ) No |
| 59. Do you monitor calves daily for signs of bronchopneumonia/respiratory disease? | ( ) Yes ( ) No |
| 60. Do you monitor adult cattle daily for signs of pneumonia/respiratory disease? | ( ) Yes ( ) No |
| 61. How often are domestic livestock transported? | ( ) Weekly  ( ) Monthly  ( ) Bimonthly  ( ) Quarterly  ( ) Annual |

62. Please, describe your vaccination program.

Please indicate the commercial name of the vaccines used to animal categories:

- Pre-weaned Calves:
- Weaned Calves:
- Heifers:
- Dry cows:
- Postpartum cows:
- Lactating cows:
