# Supplementary material for: Biosecurity practices in the dairy farms of southern Brazil
Source: Front Vet Sci. 2024 Mar 27;11:1326688. doi: 10.3389/fvets.2024.1326688 (PMC11004291; doi:10.3389/fvets.2024.1326688)
Supplement: Supplementary file 4 [file Table_3.DOCX]

**S3 – For each item, the best biosecurity measures should be identified as expected answers to obtain the maximum score (10 points) in the questionnaire.**

**PART I - Questionnaires for general risk assessment (questions 1 to 26).**

| Question | Points per question |
| --- | --- |
| 1. Is cleaning and disinfection of calf cages/bays for receiving newborn calves carried out? | Yes – 10 points  No – no points |
| 2. How many visitors (vets, milk trucks, food deliveries, etc.) enter the farm each week? | Up to 10 visitors – 10 points  11 or more visitors per week - no points |
| 3. Do you have any protocol for visitors (visitor diary, minimizing contact with animals, use of clothing provided by the farm itself such as overalls and spare boots) | Yes – 10 points  No – no points |
| 4. Do you ask your employees to avoid contact with cattle outside your property? | Yes – 10 points  No – no points |
| 5. Are there requirements that everyone always washes their hands with soap and water before handling their animals? | Yes – 10 points  No – no points |
| 6. How often animals leave and re-enter the herd (shows, embryo transfer, external clinical care with veterinarian, exhibitions, etc.): | Never – 10 points  Rarely (1-2 times a year) – no points  Sometimes (3-6 times a year) – no points  Frequently (monthly) – no points  Very often (fortnightly/weekly) – no points |
| 7. Do the animals on the farm have contact with other animals of the same species of different ages?  If **yes,** with which category? | Yes – no points  No – 10 points  Choose the animal category:  ( ) Calves  ( ) Weaned calves  ( ) Heifers  ( ) Dry cows  ( ) Lactating cows  ( ) Newly calved cows |
| 8. Do you have tractors or trucks for towing and/or transporting livestock (except disposal animals)? | Yes – 10 points  No – no points |
| 9. Does the truck or vehicle for the transportation of disposal animals (sick and male calves) enter the property? | Yes – 10 points  No – no points |
| 10. Is there a specific parking area for vehicles on the property? | Yes – 10 points  No – no points |
| 11. Is the general vehicle parking area far from the main animal housing areas? | Yes – 10 points  No – no points |
| 12. Is there a vehicle wheel disinfection system at the entrance to the property? | Yes – 10 points  No – no points |
| 13. How often are new animals introduced into the herd? | Does not occur – 10 points  Annual – no points  Monthly – no points  Weekly – no points |
| 14. Do you have a facility for isolating animals in a quarantine system? If **yes,** how long are the animals kept on average?  Are any tests carried out on the animals before introducing them into the herd? **If yes**, which one? | Yes – 10 points  No – no points  Isolation Time (days): ________________________  List tests: ________________________ |
| 15. If there are any sick animals, can they be easily isolated from the other healthy animals in the herd? | Yes – 10 points  No – no points |
| 16. Is the isolation or quarantine place for sick animals close to the premises of resident (healthy) animals? | Yes – no points  No – 10 points |
| 17. List other domestic animals residing on the farm | This question does not score |
| 18. Do you use different equipment for feeding and manure handling? | Yes – 10 points  No – no points |
| 19. Is there treatment of animal waste and/or bedding? Please mention the treatment of litter and/or manure. | Yes, both – 10 points  Yes, only from waste – 10 points  Yes, only from bed – 10 points  No – no points  Name the treatment: |
| 20. Do you do necropsy of animal dies unexpectedly?  If **yes**, please specify if there is a specific place for necropsy and who is responsible for performing this procedure. | Yes – 10 points  No – no points  Local: ________________________ |
| 21. Where is the carcass of animals that die on the farm disposed of? | In farming – no points  Used in composting system – 10 points  The animal is buried – no points  The carcass is incinerated – 10 points  Other – no points |
| 22. Do you use vaccines and medicines according to the specifications provided in the product package insert by the manufacturers? | Yes – 10 points  No – no points |
| 23. Is there a record of the temperature monitoring of the refrigerator where vaccines and medicines that need refrigeration are stored? | Yes – 10 points  No – no points |
| 24. Do you store vaccines and medicines in refrigerator to specifically for this purpose? Or does it have other purposes?  Please tick more than one alternative where applicable. | This question does not score |
| 25. What is the "age" of the refrigerator where vaccines and medicines that need refrigeration are stored? | This question does not score |
| 26. List the diseases that you would not like to occur in the animals of your herd. | This question does not score |

**PART II - Specific questionnaires for perception and risk assessment for BVDV and BoHV-1 (questions 27 to 62)**

| 27. Do you know what BVDV is? | Yes – 10 points  No – no points |
| --- | --- |
| 28. Do you know what IBR is? | Yes – 10 points  No – no points |
| 29. Do you consider your farm protected against BVDV and IBR virus? | Yes – 10 points  No – no points |
| 30. Is there concern about the occurrence of these diseases in the herd? | Yes – 10 points  No – no points |
| 31. Which category do you believe is more predisposed to BVDV and IBR virus?  Select more than one alternative if necessary. | This question does not score  Calves  Weaned calves  Heifers  Dry cows  Lactating cows  Newly calved cows  Don't know |
| 32. Have you been informed or sought information on what to do to protect your herd from BVDV and IBR virus? | Yes – 10 points I have not looked but would like more information – no points  I am not interested – no points |
| 33. Do you know what to do if BVDV and IBR virus affect an animal? | Yes – 10 points  No – no points |
| 34. Do you think you could take measures to prevent the entry and spread of these diseases on your farm? | Yes – 10 points  No – no points |
| 35. At what age is the heifer suitable for breeding on the farm? | This question does not score |
| 36. How do you is pregnancy diagnosed in your herd? (Check all that apply) | This question does not score  Manual palpation  Ultrasound  Activity monitors  No return to heat  Blood test  Milk test (presence of GAP)  None  Other:_____________ |
| 37. Do the heifers in your herd normally need assistance during calving?  If yes, how many on average? | Yes – no points  No – 10 points  How many? ____________________ |
| 38. Do the cows in your herd generally need assistance during calving?  If yes, how many on average? | Yes – no points  No – 10 points  How many? ____________________ |
| 39. Do you consider abortion a problem in your herd? | Yes – no points  No – 10 points |
| 40. Where do females give birth to their calves? | Maternity stall – 10 points  Maternity picket – no points  Free stall in contact with other animals – no points  Pasture – no points  Other. Please specify: no points  ______________________________ |
| 41. Do you realize site disinfection carried out after females abort? | Yes – 10 points  No – no points |
| 42. When an abortion occurs, do you perform any tests on the animal? | Yes – 10 points  No – no points |
| 43. Is the aborted female separated or kept from the herd? If yes, please specify. | Yes, it is separate - 10 points  No – no points |
| 44. In relation to the aborted fetus, is any biological material from the animal sent to the laboratory? | Yes – 10 points  No – no points |
| 45. Which test(s) is requested when sending the material of the aborted fetus? | This question does not score |
| 46. What is the fate of the aborted calf/fetus? | Farming – no point  Used in composting system – 10 point or  The animal is buried – no points  The carcass is incinerated – 10 point  Other – no point |
| 47. What is the occurrence of females aborting on your farm?  If **yes**, how many? | Weekly – no point  Monthly – no point  Bimonthly – no point  Quarterly – no point  Annual – no point  Does not occur – 10 points  Quantity: |
| 48. What is the main reproductive reason for cows being culled? | This question does not score  Open/ Not pregnant  Abortions  Dystocias  Metritis  Other |
| 49. How does the reproduction of females in the herd? | Herd's own breeding bulls – no point  Third party breeding bulls – no point  Artificial Insemination – 10 points  Embryo transfer (ET; IVF) – no point  Other – no point |
| 50. When you used artificial insemination (AI), is it carried out by properly trained personnel, using clean techniques and instruments? | Yes – 10 points  No – no points |
| 51. In the case of own bulls, are examinations of their reproductive efficiency and health required?  If yes, which tests? | Yes – 10 points  No – no points  Name the examinations:  ___________________________ |
| 52. Have you ever bought or still buy pregnant animals?  If **yes**, is information provided on the animal's health, history, vaccinations, etc.? Cite. | Yes, already bought – no point  Yes, I still buy – no point  No – 10 points  Cite:  ___________________________ |
| 53. Are reproductive disorders of the animals (calving difficulties, metritis, retained placenta, etc.) recorded in notebooks or computers? | Yes – 10 points  No – no points |
| 54. Which category in your perception faces the greatest challenge for respiratory diseases? | This question does not score  Calves  Weaned calves  Heifers  Dry cows  Lactating cows  Newly calved cows |
| 55. Do you record the occurrence of respiratory diseases? | Yes – 10 points  No – no points |
| 56. Do you have pre-established treatment protocols for respiratory diseases? | Yes – 10 points  No – no points |
| 57. What is the trade name of the antimicrobials used and the duration of treatment for respiratory disease? | This question does not score |
| 58. Do you use antimicrobials preventively in healthy farm animals? | Yes – no points  No – 10 points |
| 59. Do you monitor calves daily for signs of bronchopneumonia/respiratory disease? | Yes – 10 points  No – no points |
| 60. Do you monitor adult cattle daily for signs of pneumonia/respiratory disease? | Yes – 10 points  No – no points |
| 61. How often are domestic livestock transported? | Weekly – no point  Monthly – no point  Bimonthly – no point  Quarterly – no point  Annual – no point  Never – 10 points |

62. Please, describe your vaccination program - This question does not score

Please indicate the commercial name of the vaccines used to animal categories:

- Suckling Calves:
- Weaned Calves
- Pre-reproduction heifers
- Pre-calving heifers
- Dry cows
- Postpartum cows
- Lactating cows
