# Supplementary material for: Biosecurity practices in the dairy farms of southern Brazil
Source: Front Vet Sci. 2024 Mar 27;11:1326688. doi: 10.3389/fvets.2024.1326688 (PMC11004291; doi:10.3389/fvets.2024.1326688)
Supplement: Supplementary file 5 [file Table_4.DOCX]

**S4.1** Distribution of farms categorized by biosecurity level based on appropriate herd biosecurity measures.

| **Level of risk** | **Frequency %**  (n/total) | **Minimum score**  (points) | **Maximum score**  (points) | **Hit ratio (%)** |
| --- | --- | --- | --- | --- |
| Low | 0 | 450 | 500 | From 91 to 100 |
| Medium – low | 0 | 400 | 449 | From 81 to 90 |
| Medium | 0 | 376 | 399 | From 76 to 80 |
| Medium – high | 58  (40/69) | 251 | 375 | From 51 to 75 |
| High | 42  (29/69) | 126 | 250 | From 26 to 50 |
| Very high | 0 | 0 | 125 | Below 25 |

**S4.2** Degree of risk perception of dairy herd producers.

| **Level risk perception** | **Frequency %**  (n/total) | **Score**  (points) |
| --- | --- | --- |
| Low | 4.35 (3/69) | ≤30 |
| Medium | 5.8 (4/69) | 40 |
| High | 89.85 (62/69) | ≥50 |
