# Supplementary material for: Biosecurity practices in the dairy farms of southern Brazil
Source: Front Vet Sci. 2024 Mar 27;11:1326688. doi: 10.3389/fvets.2024.1326688 (PMC11004291; doi:10.3389/fvets.2024.1326688)
Supplement: Supplementary file 6 [file Table_5.DOCX]

**S5. Proportion of binary responses of biosecurity measures present/absent in herds evaluated in the biosecurity assessment questionnaire**

| **Variables** | **N (total responses)** | | | | **Response percentage (%)** | | |
| --- | --- | --- | --- | --- | --- | --- | --- |
|  |  | **Yes** | **No** | **Blank** | **Yes** | **No** | **Blank** |
| **Q1.** Is the cleaning and disinfection of cages/stalls of calves carried out to receive newborn calves? | 69 | 58 | 11 | 0 | 84.06 | 15.94 | - |
| **Q3**.  Do you have any protocols for visitors (diary visits, minimization of contact with animals, use of clothing provided by the farm itself such as overalls and boots, reserves, etc.)? | 69 | 11 | 58 | 0 | 15.94 | 84.06 | - |
| **Q4**. Do you ask your employees to avoid contact with cattle outside your property? | 69 | 10 | 59 | 0 | 14.50 | 85.50 | - |
| **Q5**. Are there requirements that everyone wash their hands with soap and water at all times before handling their animals? | 69 | 37 | 32 | 0 | 53.63 | 46.37 | - |
| **Q8.** Do you have tractors or trucks for towing and/or transporting cattle (except for disposal animals)? | 69 | 13 | 56 | 0 | 18.84 | 81.16 | - |
| **Q9.** Does the truck or vehicle for the transport of the disposal animals (sick and male calves) enter the property? | 69 | 49 | 20 | 0 | 71.01 | 28.99 | - |
| **Q10**. Is there a specific area for car parking at the property? | 69 | 19 | 48 | 2 | 27.54 | 69.56 | 2.90 |
| **Q11.** Is the vehicle parking area in general far from the main areas of accommodation of the animals? | 69 | 13 | 54 | 2 | 18.84 | 78.26 | 2.90 |
| **Q12.** Is there a vehicle wheel disinfection system at the entrance to the property? | 69 | 0 | 68 | 1 | 0 | 98.55 | 1.45 |
| **Q14.** Do you have facility for isolation of animals in quarantine system? | 69 | 41 | 25 | 3 | 59.42 | 36.23 | 4.35 |
| **Q15.** If there are any sick animals, is it possible easily to isolate them from the other healthy animals in the herd? | 69 | 42 | 26 | 1 | 60.87 | 37.68 | 1.45 |
| **Q16.** Is the isolation or quarantine site for sick animals close to the facilities of the resident (healthy) animals? | 69 | 37 | 31 | 1 | 53.62 | 44.93 | 1.45 |
| **Q18**. Is the isolation or quarantine site for sick animals close to the facilities of the resident (healthy) animals? | 69 | 15 | 54 | 0 | 21.74 | 72.26 | - |
| **Q20.** Do you do necropsy when there is an unexpected death of an animal? | 69 | 28 | 41 | 0 | 40.58 | 59.42 | - |
| **Q22.** Do you used vaccines and medicines according to the specifications provided in the product package leaflet by the manufacturers? | 69 | 69 | 0 | 0 | 100 | 0 | - |
| **Q23**. Is there any record of the temperature monitoring of the refrigerator where vaccines and medicines that need refrigeration are stored? | 69 | 12 | 56 | 1 | 17.40 | 81.15 | 1.45 |
| **Q24.** Does had a specific use for refrigerator that vaccines and medicines are stored? | 69 | 42 | 27 | 0 | 60.87 | 39.13 | - |

| *Q27 a Q34, Q39, Q54 – About the risk perception* | | | | | | | |
| --- | --- | --- | --- | --- | --- | --- | --- |
| **Q27.** Do you know BVD? | 69 | 61 | 8 | 0 | 88.41 | 11.59 | - |
| **Q28.** Do you know IBR? | 69 | 61 | 8 | 0 | 88.41 | 11.59 | - |
| **Q29.** Do you consider your farm to be protected against BVD and IBR viruses? | 68¹ | 33 | 35 | 1 | 47.83 | 50.72 | 1.45 |
| **Q30.** Do you worry about the occurrence of these diseases in your herd? | 67¹ | 65 | 2 | 2 | 94.20 | 2.90 | 2.90 |
| **Q31.** Which category do you believe is more predisposed to BVD and IBR viruses?  (Select more than one alternative if necessary) | 69 |  |  |  |  |  |  |
| Pre-weaned and Weaned |  | 43 | - | - | 62.31 | - | - |
| Lactating cows |  | 14 | - | - | 20.29 | - | - |
| Heifers |  | 10 | - | - | 14.50 | - | - |
| Dry cows |  | 1 | - | - | 1.45 | - | - |
| Fresh cows |  | 1 | - | - | 1.45 | - | - |
| **Q32.** Have you been informed or sought information on what to do to protect your herd from the BVDV virus and the IBR virus? | 69 |  |  |  |  |  |  |
| Yes |  | 50 | - | - | 72.46 | - | - |
| I haven't looked, but I'd like more information |  | 19 | - | - | 27.54 | - | - |
| No interest |  | 0 | - | - | - | - | - |
| **Q33.** Do you know what to do if an animal is infected with BVDV and the IBR virus? | 69 | 60 | 8 | 1 | 86.95 | 11.60 | 1.45 |
| **Q34.** Do you think you could take measures to prevent the entry and spread of these diseases on your property? | 69 | 66 | 3 | 0 | 95.65 | 4.35 | - |
| **Q37**. Do the heifers in your herd normally need assistance during calving?  If yes, how many on average? | 69 | 22 | 47 | - | 31.88 | 68.11 | - |
| **Q38.** Do the cows in your herd usually need assistance during calving?  If yes, how many on average? | 69 | 18 | 51 | - | 26.08 | 73.91 | - |
| **Q39.** Do you consider abortion a problem in your flock? | 67¹ | 30 | 35 | 2 | 44.77 | 52.23 | 2.98 |
| **Q41**. Do you do disinfection on site after females abort? | 69 | 10 | 59 | - | 14.5 | 85.50 | - |
| **Q42**. When an abortion occurs, do you do any tests performed on the animal? | 69 | 8 | 61 | - | 11.59 | 88.40 | - |
| **Q43**. Do you used to separate aborted female from the herd? | 69 | 14 | 55 | - | 20.28 | 79.71 | - |
| **Q44.** Do you send any biological material of fetus to laboratory? | 69 | 2 | 67 | - | 2.89 | 97.10 | - |
| **Q50**. If artificial insemination (AI) is used, do you have trained staff? To use clean techniques and instruments? | 69 | 68 | 1 | - | 98.55 | 1.45 | - |
| **Q51.** In the case of own bulls, are tests required on the reproductive efficiency and health of this bull?  If yes, which tests? | 69 | 5 | 0 | 64 | 7.24 | 0 | 92.76 |
| **Q52.** Do you have to buy pregnant animals? | 69 | 29 | 40 | - | 42.02 | 57.97 | - |
| **Q53.** Are reproductive disorders of the animals (calving difficulties, metritis, retained placenta, etc.) recorded in notebooks or computers? | 68 | 65 | 3 | 1 | 94.20 | 4.34 | - |
| **Q54.** Which category do you think faces the biggest challenge for respiratory diseases? | 69 |  |  |  |  |  |  |
| Pre-weaned |  | 52 | - | - | 75.36 | - | - |
| Lactating cows |  | 12 | - | - | 17.39 | - | - |
| Fresh cows |  | 2 | - | - | 2.90 | - | - |
| Blank answers |  | 2 | - | - | 2.90 | - | - |
| Weaned until 12 mo |  | 1 | - | - | 1.45 | - | - |
| **Q55.** Do you recorded occurrence of respiratory diseases in the herd? | 69 | 63 | 6 | - | 91.30 | 8.69 | - |
| **Q56.** Do you have pre-established treatment protocols for respiratory diseases?  If yes, which one? | 69 | 40 | 29 | - | 57.97 | 42.02 | - |
| **Q58**. Did you use antimicrobial as prevent in healthy farm animals? | 69 | 10 | 59 | - | 14.5 | 85.50 | - |
| **Q59.** Did you monitor calves daily for signs of bronchopneumonia/respiratory disease? | 69 | 64 | 4 | 1 | 92.75 | 5.8 | 1.45 |
| **Q60**. Did you monitor adult cattle daily for signs of pneumonia/respiratory disease? | 69 | 66 | 3 | - | 95.65 | 4.35 | - |
| **Q62.** Do you have a vaccination schedule for reproductive and respiratory diseases? | 64¹ | 62 | 2 | 5 | 89.85 | 2.90 | 7.25 |

¹Reduced sample size due to blank answers in the questionnaire
